# Factors Associated With Falls Among the Elderly With Type 2 Diabetes in Urban Shanghai: A Cross‐Sectional Study

**DOI:** 10.1155/jdr/8624656

**Published:** 2026-09-24

**Authors:** Zhenping Lu, Hui Gao, Huiyao Zhu, Dong Chen, Yinzhu Zhuang, Lei Zhang, Li Yu, Qinghua Xia, Yu Jiang

**Affiliations:** ^1^ The Shanghai Changning District Center for Disease Control and Prevention (Changning District Health Supervision Institute), Shanghai, China

**Keywords:** associated factors, elderly, falls, Type 2 diabetes mellitus

## Abstract

**Aim:**

This study is aimed at identifying the factors associated with falls among community‐dwelling elderly individuals with Type 2 diabetes mellitus (T2DM) in urban Shanghai, China.

**Methods:**

The study analyzed data from 842 diabetic patients aged 60 years or older. Information about associated factors and main outcome measures was collected using structured questionnaires. Chi‐square tests and binary logistic regression were used to identify factors associated with falls.

**Results:**

Among the participants, 239 (28.4%) experienced at least one fall and 41 (17.2%) of these fallers had recurrent falls in the past 1 year. Univariate analysis identified a range of potential factors for falls, including physical impairments (e.g., dizziness, fatigue, unsteadiness), psychological factors (e.g., depression, activity restriction due to fear of falling), and adverse lifestyle factors (e.g., inactivity, poor sleep, frequent nocturia) (*p* < 0.05). Logistic regression analysis revealed that balance test score (OR = 1.687, 95% CI: 1.162–2.447, *p* = 0.006), PHQ‐9 depression category (OR = 1.663, 95% CI: 1.028–2.691, *p* = 0.038) and activity restriction due to fear of falling (OR = 2.503, 95% CI: 1.752–3.575, *p* < 0.001) were independently associated with falls. For recurrent falls, activity restriction due to fear of falling remained significantly associated across models (OR range: 2.045–2.165, *p* < 0.05).

**Conclusion:**

Falls were common among elderly diabetic residents in urban Shanghai, and multiple factors were identified as potential correlates. Our findings support the use of multidomain screening to identify high‐risk individuals for falls and to inform targeted assessment approaches, warranting further prospective investigations.

## 1. Introduction

Falls represent a pervasive and serious public health challenge among older populations worldwide. Each year, more than one‐quarter of individuals aged 65 or older sustain at least one fall [1], an event that not only undermines their physical autonomy and well‐being but also contributes to elevated rates of illness, mortality, and healthcare expenditure [2, 3]. Meanwhile, China faces the heaviest diabetes burden globally. National surveillance data from 2015 to 2018 indicate that the crude incidence of diabetes among middle‐aged and older Chinese adults reached approximately 3920 per 100,000 person‐years, with a pronounced upward trajectory over this period [4]. A fall was defined as “an unexpected event in which the participant comes to rest on the ground, floor, or lower level” [5]. Accumulating evidence indicates that older adults with T2DM face a higher fall risk than those without [6–8]. Several studies have identified a range of correlates of falls in this population, including age, polypharmacy, insulin therapy, peripheral neuropathy, and impaired balance [9–11]. Nevertheless, the role of certain factors remains inconclusive, and the vast majority of existing evidence originates from Western settings [12–14]. Given the paucity of research on fall‐related issues among older Chinese adults with diabetes, further population‐based investigations are warranted to explore modifiable factors associated with falls in this population. To address this gap, the present study aimed to estimate the prevalence of falls and identify its associated factors among community‐dwelling older adults with T2DM in urban Shanghai, China.

## 2. Methods

### 2.1. Study Design and Participants

**Figure 1:**
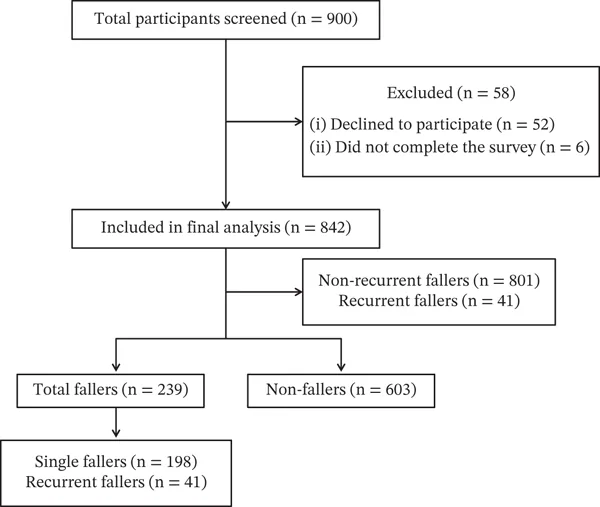
Flowchart of participant selection for the study.

This cross‐sectional study was conducted in Changning District, Shanghai, China, between May and June 2024. The inclusion criteria were as follows: (1) age ≥ 60 years; (2) currently registered as a Type 2 diabetes patient in the community; (3) ability to communicate normally and walk independently; and (4) voluntary participation in the survey with signed informed consent. The sample size was determined using the formula: ${\mu^{2}}_{\alpha /2}\pi \left(1-\pi \right)/\delta^{2}$, where *μ*_*α*/2_ = 1.96 (two‐sided *α* = 0.05), *π* = 0.30 was the estimated fall prevalence based on previous studies, and the relative error was set at 10%, giving an absolute error *δ* = 0.03. The calculated sample size was 896, which was rounded up to 900 to allow for missing or incomplete data. The sampling frame consisted of community‐managed elderly T2DM patients aged ≥ 60 years registered in the Changning District Chronic Disease Health Management System (total population: 28,300 individuals). Using simple random sampling with a fixed random seed of 123456, 900 diabetic patients were initially selected from this frame. Trained community physicians contacted the selected individuals by telephone to explain the study objectives and invite them to participate. Of these, 52 individuals declined to participate, mainly due to time constraints or lack of interest. Six participants did not complete the survey, due to partial completion of the questionnaire or early withdrawal during the assessment. Ultimately, 842 elderly patients with T2DM were included in the final analysis (response rate: 93.6%). The participant selection process is illustrated in Figure 1.

### 2.2. Data Collection and Definitions

Data were collected using self‐designed questionnaires (Supporting Information 1: Table S1) through face‐to‐face interviews. The collected information included: (1) demographic and socioeconomic characteristics (gender, age, marital status, educational level, cohabitation status, self‐care ability in daily living, and annual household income); (2) clinical and comorbidity‐related factors (diabetes duration, glycemic control status, and history of diabetic complications, including nephropathy, retinopathy, neuropathy, and diabetic foot disease, other chronic disease); (3) medication‐related factors (use of insulin, thiazolidinedione, sulfonylurea, sedative‐hypnotic, or psychotropic medications); (4) physical function and mobility factors (dizziness, fatigue, subjective unsteadiness, and limb paresthesia); (5) psychological and cognitive factors (activity restriction due to fear of falling); and (6) lifestyle and behavioral factors (use of walking aid, smoking status, average daily sleep duration, number of nighttime awakenings, exercise habits, and dietary habits).

In this study, T2DM was defined as meeting at least one of the following criteria: (1) a prior physician‐confirmed diagnosis of T2DM; or (2) the fasting plasma glucose level ≥ 7.0 mmol/L during medical examination. Fall history was assessed by asking participants whether they had experienced any falls in the past 12 months, and if so, the number of falls, along with details on location, cause, nature of injury, and postfall management. Based on the reported number of falls, participants were categorized as non‐fallers (0 falls), single fallers (1 fall), or recurrent fallers (≥ 2 falls). For group comparisons, non‐recurrent fallers were defined as participants who experienced 0 or 1 fall during the past year. Diabetes duration was categorized into three groups: < 5 years, 5–10 years, and > 10 years [15]. Polypharmacy was defined as the use of four or more medications [16, 17]. Body weight status of the study participants was categorized into underweight (BMI < 18.5 kg/m^2^), normal weight (18.5 kg/m^2^ ≤ BMI < 24 kg/m^2^), overweight (24 kg/m^2^ ≤ BMI <28 kg/m^2^), and obesity (BMI ≥ 28 kg/m^2^) [18]. Central obesity was defined as a waist circumference ≥ 90 cm for men and ≥ 80 cm for women [19]. A calf circumference of less than 34 cm in men and less than 33 cm in women was defined as an increased risk of sarcopenia [20].

### 2.3. Measurement Methods

Height and weight were measured using calibrated stadiometers and scales. Calf circumference was measured at the maximal girth of the calf using a nonelastic tape, and waist circumference was measured at the midpoint between the lower costal margin and the iliac crest, both recorded to the nearest 0.1 cm. Balance performance was assessed using the X16 balance testing scale (X16‐BS) [21], with total scores ranging from 0 to 20. Balance performance was dichotomized into intact (score ≥ 17) versus impaired (score < 17). Depressive symptom severity was assessed using the Patient Health Questionnaire‐9 (PHQ‐9) [22]. Participants were dichotomized into those with depressive symptoms (score: 5–27) and those without (score: 0–4). The Chinese version of the 16‐item Falls Efficacy Scale International (FES‐I) was administered to participants [23, 24]. Each item was rated from 1 (*not at all concerned*) to 4 (*very concerned*), with total scores ranging from 16 to 64. Based on established criteria [25], participants were classified into three groups: low concern (16–19), moderate concern (20–27), and high concern (28–64). Cognitive impairment was screened using the Montreal Cognitive Assessment (MoCA), applying the following classification: normal cognition (score ≥ 23) and cognitive impairment (score ≤ 22) [26, 27]. The MoCA was used under authorization from MoCA Test Inc. for this noncommercial academic research and is not reproduced in this manuscript.

### 2.4. Statistical Analysis

Statistical analysis was performed using IBM SPSS Statistics (Version 22.0). Descriptive data was reported as frequencies, and percentages. For comparisons of categorical variables, Pearson′s chi‐square test was used when all expected cell counts were ≥ 5. When this assumption was violated, Fisher′s exact test was used for 2 × 2 tables and the Fisher–Freeman–Halton exact test for R × C tables. Multicollinearity was assessed using the variance inflation factor (VIF), with VIF values for all variables ranging from 1.0 to 1.5. Binary logistic regression analyses were performed to analyze factors associated with falls (fallers vs. nonfallers) and recurrent falls (recurrent fallers vs. nonrecurrent fallers). Variables were selected for inclusion based on clinical relevance and prior literature, rather than on statistical significance alone. For falls, three hierarchical models were constructed. For recurrent falls, three models were also constructed, with the number of factors strictly limited to four due to the limited number of recurrent fall events. All variables were entered simultaneously using the enter method and the results were presented as odds ratios (ORs) with 95% confidence intervals (CIs).

Missing data for continuous variables [PHQ‐9 (*n* = 30, 3.6%), FES‐I (Ch) (*n* = 17, 2.0%), MoCA (*n* = 21, 2.5%), and BMI (*n* = 9, 1.1%)] were imputed using the mean of the observed values prior to categorization into PHQ‐9 depression category, fall efficacy category, presence of cognitive impairment, and body weight status, respectively. For the categorical variable (total annual household income in the past year: *n* = 31, 3.7%), mode imputation was applied. All results were considered statistically significant at two‐sided *p* value < 0.05.

## 3. Results

### 3.1. Description of the Participants

This study included 842 elderly diabetic patients, consisting of 333 males and 509 females. The mean age of the total population was 72.2 ± 6.2 years, with more than half (54.6%) in the 70–79 years age group. 11.5% of participants reported living alone, and 92.6% preserved their self‐care ability in daily living. Among clinical and comorbidity‐related factors, 65.6% had a diabetes duration of ≥ 5 years, 13.4% had a history of diabetic complications, and 27.1% were taking ≥ 4 medications. Among psychological and cognitive factors, 11.6% had depressive symptoms, 35.0% exhibited cognitive impairment, 24.7% restricted their activities due to fear of falling and 46.8% reported high concern about falling. For lifestyle and behavioral factors, 81.8% were physically inactive, 10.5% were smokers, and 52.3% had a nighttime sleep duration of < 7 h. A number of baseline characteristics exhibited significant gender disparities, including age, educational level, status of living, presence of multimorbidity, presence of unsteadiness, presence of central obesity, smoking, and nighttime sleep duration (all *p* < 0.05) (Table 1). Other baseline characteristics and potential factors, overall and by sex, are shown in Table S2.

**Table 1:** Baseline characteristics and potential associated factors for falls among the elderly with T2DM, overall and by sex.

Variables	Overall (*N* = 842)	Male (*N* = 333)	Female (*N* = 509)	*χ* ^2^	*p*
Age (years)				22.251	**< 0.001**
60–69	283 (33.6%)	88 (26.4%)	195 (38.3%)		
70–79	460 (54.6%)	188 (56.5%)	272 (53.4%)		
≥ 80	99 (11.8%)	57 (17.1%)	42 (8.3%)		
Educational level				20.143	**< 0.001**
Primary school or below	72 (8.6%)	26 (7.8%)	46 (9%)		
Junior high school	341 (40.5%)	118 (35.4%)	223 (43.8%)		
Senior high school or technical secondary school	307 (36.5%)	119 (35.7%)	188 (36.9%)		
Junior college or above	122 (14.5%)	70 (21.0%)	52 (10.2%)		
Living alone				20.207	**< 0.001**
No	745 (88.5%)	315 (94.6%)	430 (84.5%)		
Yes	97 (11.5%)	18 (5.4%)	79 (15.5%)		
Self‐care in daily living				0.020	0.888
No	62 (7.4%)	24 (7.2%)	38 (7.5%)		
Yes	780 (92.6%)	309 (92.8%)	471 (92.5%)		
Duration of diabetes (years)				1.245	0.537
< 5	290 (34.4%)	108 (32.4%)	182 (35.8%)		
5–10	248 (29.5%)	104 (31.2%)	144 (28.3%)		
> 10	304 (36.1%)	121 (36.3%)	183 (36.0%)		
History of diabetic complications				0.468	0.494
No	729 (86.6%)	285 (85.6%)	444 (87.2%)		
Yes	113 (13.4%)	48 (14.4%)	65 (12.8%)		
Presence of multimorbidity (≥ 5 conditions)				14.568	**< 0.001**
No	685 (81.4%)	292 (87.7%)	393 (77.2%)		
Yes	157 (18.6%)	41 (12.3%)	116 (22.8%)		
Polypharmacy (≥ 4 medications)				0.587	0.444
No	614 (72.9%)	238 (71.5%)	376 (73.9%)		
Yes	228 (27.1%)	95 (28.5%)	133 (26.1%)		
Subjective unsteadiness				4.294	**0.038**
No	583 (69.2%)	217 (65.2%)	366 (71.9%)		
Yes	259 (30.8%)	116 (34.8%)	143 (28.1%)		
Central obesity				80.380	**< 0.001**
No	317 (37.6%)	187 (56.2%)	130 (25.5%)		
Yes	525 (62.4%)	146 (43.8%)	379 (74.5%)		
PHQ‐9 depression category					
Without depressive symptoms	744 (88.4%)	302 (90.7%)	442 (86.8%)	2.907	0.088
With depressive symptoms	98 (11.6%)	31 (9.3%)	67 (13.2%)		
Activity restriction due to fear of falling				1.823	0.177
No	634 (75.3%)	259 (77.8%)	375 (73.7%)		
Yes	208 (24.7%)	74 (22.2%)	134 (26.3%)		
Fall efficacy category				2.593	0.273
Low concern	213 (25.3%)	92 (27.6%)	121 (23.8%)		
Moderate concern	235 (27.9%)	96 (28.8%)	139 (27.3%)		
High concern	394 (46.8%)	145 (43.5%)	249 (48.9%)		
Presence of cognitive impairment				1.640	0.200
Normal cognition	547 (65.0%)	225 (67.6%)	322 (63.3%)		
Cognitive impairment	295 (35.0%)	108 (32.4%)	187 (36.7%)		
Habitual exercise				0.074	0.785
No	689 (81.8%)	271 (81.4%)	418 (82.1%)		
Yes	153 (18.2%)	62 (18.6%)	91 (17.9%)		
Smoking				103.683	**< 0.001**
No	754 (89.5%)	254 (76.3%)	500 (98.2%)		
Yes	88 (10.5%)	79 (23.7%)	9 (1.8%)		
Nighttime sleep duration ≥ 7 h				3.911	**0.048**
No	440 (52.3%)	160 (48.0%)	280 (55.0%)		
Yes	402 (47.7%)	173 (52.0%)	229 (45.0%)		

*Note:* History of diabetic complications was defined as the presence of any of the following: diabetic nephropathy, diabetic eye disease, diabetic neuropathy, or diabetic foot disease. Bolded values denote variables with statistically significant gender‐based differences (between males and females).

### 3.2. Univariate Analysis of Potential Factors Associated With Falls

In total, 239 of the 842 participants (28.4%) experienced at least one fall in the past year. Among these fallers, 41 individuals (17.2%) had recurrent falls, accounting for 4.9% of the total study population. The comparison of potential factors across different fall categories was presented in Table 2 and Table S3. Fall status was significantly associated with a range of factors, encompassing the lack of self‐care ability, physical impairments (frequent dizziness, persistent fatigue, subjective unsteadiness, limb paresthesia, poor balance), psychological factors (depression, activity restriction due to fear of falling, concern about falling), and lifestyle factors (inactivity, poor sleep, frequent nocturia, use of walking aids) (all *p* < 0.05). Factors significantly associated with recurrent falls included history of diabetic complications, frequent dizziness, persistent fatigue, subjective unsteadiness, limb paresthesia, depression, activity reduction due to fear of falling, frequent nocturia, and use of walking aids (all *p* < 0.05) (Table 2). Other variables were not significantly associated with either falls or recurrent falls (all *p* > 0.05) (Supporting Information 1: Table S3).

**Table 2:** Comparison of potential associated factors across different fall categories.

Variables	Total fallers (*N* = 239)	Nonfallers (*N* = 603)	*χ* ^2^	*p*	Nonrecurrent fallers (*N* = 801)	Recurrent fallers (*N* = 41)	*χ* ^2^	*p*
Demographic and socioeconomic characteristics								
Independence in activities of daily living			**4.692**	**0.030**			‐^a^	0.113
No	25 (10.5%)	37 (6.1%)			56 (7.0%)	6 (14.6%)		
Yes	214 (89.5%)	566 (93.9%)			745 (93.0%)	35 (85.4%)		
Clinical and comorbidity‐related factors								
History of diabetic complications			1.220	0.269			**4.464**	**0.035**
No	202 (84.5%)	527 (87.4%)			698 (87.1%)	31 (75.6%)		
Yes	37 (15.5%)	76 (12.6%)			103 (12.9%)	10 (24.4%)		
Physical function and mobility factors								
Frequent dizziness			**6.683**	**0.010**			**5.349**	**0.021**
No	162 (67.8%)	461 (76.5%)			599 (74.8%)	24 (58.5%)		
Yes	77 (32.2%)	142 (23.5%)			202 (25.2%)	17 (41.5%)		
Persistent fatigue			**4.819**	**0.028**			**20.033**	**< 0.001**
No	201 (84.1%)	540 (89.6%)			714 (89.1%)	27 (65.9%)		
Yes	38 (15.9%)	63 (10.4%)			87 (10.9%)	14 (34.1%)		
Subjective unsteadiness			**34.538**	**< 0.001**			**15.613**	**< 0.001**
No	130 (54.4%)	453 (75.1%)			566 (70.7%)	17 (41.5%)		
Yes	109 (45.6%)	150 (24.9%)			235 (29.3%)	24 (58.5%)		
History of limb paresthesia			**3.905**	**0.048**			**11.430**	**0.001**
No	140 (58.6%)	397 (65.8%)			521 (65.0%)	16 (39%)		
Yes	99 (41.4%)	206 (34.2%)			280 (35.0%)	25 (61%)		
Balance test score			**19.333**	**< 0.001**			2.451	0.117
≥ 17	163 (68.2%)	495 (82.1%)			630 (78.7%)	28 (68.3%)		
< 17	76 (31.8%)	108 (17.9%)			171 (21.3%)	13 (31.7%)		
Psychological and cognitive factors								
PHQ‐9 depression category			**13.096**	**< 0.001**			‐^a^	**0.045**
Without depressive symptoms	196 (82.0%)	548 (90.9%)			712 (88.9%)	32 (78.0%)		
With depressive symptoms	43 (18.0%)	55 (9.1%)			89 (11.1%)	9 (22.0%)		
Activity restriction due to fear of falling			**45.260**	**< 0.001**			**6.509**	**0.011**
No	142 (59.4%)	492 (81.6%)			610 (76.2%)	24 (58.5%)		
Yes	97 (40.6%)	111 (18.4%)			191 (23.8%)	17 (41.5%)		
Fall efficacy category			**16.244**	**< 0.001**			3.225	0.199
Low concern	46 (19.2%)	167 (27.7%)			207 (25.8%)	6 (14.6%)		
Moderate concern	55 (23.0%)	180 (29.9%)			224 (28.0%)	11 (26.8%)		
High concern	138 (57.7%)	256 (42.5%)			370 (46.2%)	24 (58.5%)		
Lifestyle and behavioral factors								
Habitual exercise			**6.070**	**0.014**			1.035	0.309
No	208 (87%)	481 (79.8%)			653 (81.5%)	36 (87.8%)		
Yes	31 (13%)	122 (20.2%)			148 (18.5%)	5 (12.2%)		
Nighttime sleep duration ≥ 7 h			**4.023**	**0.045**			0.209	0.648
No	138 (57.7%)	302 (50.1%)			420 (52.4%)	20 (48.8%)		
Yes	101 (42.3%)	301 (49.9%)			381 (47.6%)	21 (51.2%)		
Frequent nocturia (≥ 2 times/night)			**4.765**	**0.029**			**4.959**	**0.026**
No	125 (52.3%)	365 (60.5%)			473 (59.1%)	17 (41.5%)		
Yes	114 (47.7%)	238 (39.5%)			328 (40.9%)	24 (58.5%)		
Daily use of walking aids			**3.892**	**0.049**			**-** ^**a**^	**0.018**
No	216 (90.4%)	568 (94.2%)			750 (93.6%)	34 (82.9%)		
Yes	23 (9.6%)	35 (5.8%)			51 (6.4%)	7 (17.1%)		

*Note:* The bolded texts represent values (including χ^2^ and *p* values) with statistically significant differences between fallers and nonfallers, as well as between recurrent fallers and nonrecurrent fallers.

^a^Fisher′s exact test was used for variables with expected cell counts < 5.

### 3.3. Multivariable Analysis of Potential Factors Associated With Falls

Table 3 presents the results of the multivariable logistic regression analysis for factors associated with falls. In the fully adjusted model (Model 3), balance test score (OR = 1.687, 95% CI: 1.162–2.447, *p* = 0.006), PHQ‐9 depression category (OR = 1.663, 95% CI: 1.028–2.691, *p* = 0.038) and activity restriction due to fear of falling (OR = 2.503, 95% CI: 1.752–3.575, *p* < 0.001) were significantly associated with falls in the elderly with T2DM. The remaining variables in the model did not reach statistical significance (Table 3).

**Table 3:** Binary logistic regression analysis of factors associated with falls in the elderly with T2DM (*N* = 842).

Variables	Model 1		Model 2		Model 3	
	Adjusted OR (95% CI)	*p*	Adjusted OR (95% CI)	*p*	Adjusted OR (95% CI)	*p*
Gender						
Male	Ref		Ref		Ref	
Female	1.268 (0.925–1.738)	0.140	1.257 (0.912–1.733)	0.162	1.133 (0.814–1.577)	0.459
Age (years)						
60–69	Ref		Ref		Ref	
70–79	1.178 (0.842–1.649)	0.339	1.070 (0.758–1.509)	0.701	0.918 (0.642–1.312)	0.637
≥ 80	1.173 (0.696–1.978)	0.550	0.980 (0.568–1.692)	0.944	0.743 (0.419–1.320)	0.311
Duration of Diabetes (years)						
<5	Ref		Ref		Ref	
5–10	0.970 (0.661–1.424)	0.876	0.983 (0.667–1.450)	0.932	0.983 (0.659–1.466)	0.934
> 10	1.141 (0.794–1.640)	0.475	1.168 (0.809–1.688)	0.406	1.181 (0.808–1.725)	0.390
History of diabetic complications						
No	Ref		Ref		Ref	
Yes	1.197 (0.775–1.849)	0.418	1.186 (0.763–1.845)	0.448	0.930 (0.578–1.495)	0.763
Polypharmacy (≥ 4 medications)						
No	Ref		Ref		Ref	
Yes	1.189 (0.849–1.666)	0.313	1.153 (0.818–1.626)	0.416	1.061 (0.743–1.515)	0.743
Balance test score	—					
≥ 17			Ref		Ref	
< 17			2.117 (1.486–3.017)	**< 0.001**	1.687 (1.162–2.447)	**0.006**
Increased risk of sarcopenia	—					
No			Ref		Ref	
Yes			0.835 (0.603–1.157)	0.278	0.771 (0.551–1.079)	0.129
Presence of cognitive impairment	—					
Normal cognition			Ref		Ref	
Cognitive impairment			0.934 (0.676–1.292)	0.682	0.842 (0.601–1.180)	0.317
PHQ‐9 depression category	—		—			
Without depressive symptoms					Ref	
With depressive symptoms					1.663 (1.028–2.691)	**0.038**
Activity restriction due to fear of falling	—		—			
No					Ref	
Yes					2.503 (1.752–3.575)	**< 0.001**
Fall efficacy category	—		—			
Low concern					Ref	
Moderate concern					1.014 (0.641–1.604)	0.953
High concern					1.457 (0.959–2.216)	0.078

*Note:* Model 1: adjusted for gender, age, duration of diabetes, history of diabetic complications and polypharmacy. Model 2: adjusted for gender, age, duration of diabetes, history of diabetic complications, polypharmacy, balance test score, increased risk of sarcopenia and presence of cognitive impairment. Model 3: adjusted for gender, age, duration of diabetes, history of diabetic complications, polypharmacy, balance test score, increased risk of sarcopenia, presence of cognitive impairment, PHQ‐9 depression category, activity restriction due to fear of falling and fall efficacy category. The bolded texts represent the *p* values of variables that are significantly associated with falls in the models.

Table 4 summarizes the associations between potential factors and recurrent falls across three logistic regression models. Activity restriction due to fear of falling remained significantly associated with recurrent falls in Model 2 (OR = 2.165, 95% CI: 1.107–4.235, *p* = 0.024) and Model 3 (OR = 2.045, 95% CI: 1.057–3.955, *p* = 0.034) (Table 4).

**Table 4:** Binary logistic regression analysis of factors associated with recurrent falls in the elderly with T2DM (*N* = 842).

Variables	Model 1		Model 2		Model 3	
	Adjusted OR (95% CI)	*p*	Adjusted OR (95% CI)	*p*	Adjusted OR (95% CI)	*p*
Gender					—	
Male	Ref		Ref			
Female	0.943 (0.492–1.806)	0.859	0.838 (0.433–1.622)	0.600		
Age (years)					—	
60–69	Ref		Ref			
70–79	0.723 (0.358–1.458)	0.364	0.644 (0.315–1.313)	0.226		
≥ 80	1.220 (0.469–3.179)	0.683	1.027 (0.388–2.717)	0.957		
Duration of diabetes (years)			—			
< 5	Ref				Ref	
5–10	0.965 (0.409–2.280)	0.935			0.965 (0.408–2.286)	0.936
> 10	1.378 (0.643–2.952)	0.410			1.366 (0.641–2.910)	0.419
History of diabetic complications			—			
No	Ref				Ref	
Yes	2.076 (0.972–4.433)	0.059			1.696 (0.767–3.747)	0.192
PHQ‐9 depression category	—					
Without depressive symptoms			Ref		Ref	
With depressive symptoms			2.032 (0.917–4.505)	0.081	1.551 (0.672–3.581)	0.304
Activity restriction due to fear of falling	—					
No			Ref		Ref	
Yes			2.165 (1.107–4.235)	**0.024**	2.045 (1.057–3.955)	**0.034**

*Note:* Model 1: adjusted for gender, age, duration of diabetes and history of diabetic complications. Model 2: adjusted for gender, age, PHQ‐9 depression category and activity restriction due to fear of falling. Model 3: adjusted for duration of diabetes, history of diabetic complications, PHQ‐9 depression category and activity restriction due to fear of falling. The bolded texts represent the *p* values of variables that are significantly associated with recurrent falls in the models.

## 4. Discussion

In this study, we systematically examined the prevalence of falls and its associated factors among older adults with T2DM. Our findings reveal a considerable fall burden in this urban Shanghai population, with 28.4% of participants reporting at least one fall and 17.2% of those who fell experiencing recurrent falls within the preceding year. The independent associations observed for balance impairment, depressive symptoms, and activity restriction due to fear of falling are consistent with the hypothesis that falls in older adults arise from a complex interplay of physical, psychological, and behavioral determinants.

The fall prevalence observed in our study exceeded that documented in comparable populations from Taiwan (19.8%) [28], Guangzhou (21.96%) [29] and Hong Kong (23.3%) [30]. Conversely, certain international studies have reported higher figures, including a rate of 39% among diabetic individuals over 65 years of age in London [13, 31]. This could be attributed to differences in demographic characteristics and study periods. Of particular note, the fall and recurrent fall rates in our diabetic sample were higher than those reported in the majority of previous studies involving general older population [32–34]. Given the profound implications for patients, their families, and the broader healthcare system, this elevated fall risk in the diabetic elderly population calls for prioritized attention. Therefore, targeted investigations into fall‐related factors among community‐dwelling older adults with diabetes in urban Shanghai are urgently warranted.

In univariable analyses, falls and recurrent falls were significantly associated with self‐perceived physical function‐related factors, including dizziness, fatigue, subjective unsteadiness, limb paresthesia, and activity restriction due to fear of falling. These findings are supported by previous research demonstrating that factors such as dizziness, fatigue, and activity restriction due to fear of falling are independent predictors of accidental falls in elderly patients with diabetes [35–37]. However, it is important to recognize that some of these factors may represent consequences rather than antecedents of falls. For instance, activity restriction may arise from fear following a prior fall, and subjective unsteadiness or fatigue could be exacerbated by reduced physical activity after a fall event. Therefore, the potential for reverse causality should be carefully considered when interpreting these associations. In addition, several modifiable behavioral factors were significantly associated with falls, including physical inactivity, short sleep duration, frequent nocturia, and use of walking aids. These findings are consistent with previous literature [36, 38–40]. For example, a large population‐based study using data from the Korean Community Health Survey showed that more frequent nocturia was associated with progressively higher odds of falls [40]. In summary, our findings align with the World Falls Guidelines [1], supporting the multifactorial nature of falls and the implementation of multidomain screening in community‐dwelling older adults with diabetes.

Multivariate regression analysis identified balance test score, depressive symptoms (assessed by the PHQ‐9) and activity restriction due to fear of falling as factors independently associated with falls. Previous studies have shown that balance impairment contributes to increased fall risk in older adults with Type 2 diabetes [10, 41, 42]. This may be explained by the fact that diabetes affects balance primarily through multisystem impairments, including peripheral neuropathy, visual dysfunction, and vestibular impairment, which collectively compromise proprioception, sensory feedback, and postural control, thereby increasing the risk of falls [10, 43, 44]. Furthermore, depressive symptoms warrant integrated consideration, as they have been consistently associated with falls in previous studies [28, 36, 45], which aligns with our findings. Depression may increase fall risk in older adults with diabetes through multiple pathways [45–48], among which the most frequently cited involves cognitive deficits that affect attention, executive function, and processing speed, thereby contributing to an elevated risk of falls [49]. Consistent with the ADA′s 4Ms framework for geriatric diabetes care [50], fall screening in older adults with diabetes should extend beyond physical function to include psychological assessment for depression and cognitive status. Several factors have been reported to be associated with falls in individuals with diabetes, including gender, age, diabetes duration, history of complications, polypharmacy, sarcopenia, and cognitive impairment [28, 29, 45, 51–54]. However, none of these factors reached statistical significance in our sample. These discrepancies may be attributed to differences in study populations and the relatively low prevalence of certain factors in our sample, which limited statistical power to detect modest associations. In addition, variations in variable definitions and measurement tools across studies may have contributed to the inconsistent findings. Notably, our study identified activity restriction due to fear of falling as a significant correlate of recurrent falls. Previous research has linked recurrent falls in older adults with diabetes to various factors, such as gait and balance problems, hypotension, polypharmacy, and HbA_1c_ level [30, 55]. However, research specifically focusing on recurrent falls in this population remains limited in China, highlighting the need for further investigation. Collectively, our findings support the integration of physical, psychological, and behavioral assessments into fall screening strategies for older adults with diabetes.

Our study revealed both the incidence of falls and their associated factors among elderly diabetic patients in community. A key strength of this study is the integrated application of multiple specialized questionnaires to identify various factors associated with falls among elderly diabetic patients, providing valuable insights for the development of targeted fall screening strategies. However, the limitations in this study should also be acknowledged. First, the retrospective assessment of fall history and self‐reported complication data may have introduced recall bias. Second, the temporal relationship between the examined factors and falls could not be established owing to the cross‐sectional design of this study. Third, this study was conducted in a single urban district of Shanghai. The findings may not be directly generalized to older adults with T2DM in other regions. Fourth, we did not collect objective glycemic control measures (e.g., HbA_1c_) or detailed information on hypoglycemia‐prone therapies or hypoglycemic episodes, which may limit the disease‐specific interpretation of our findings. Given these constraints, large‐scale, multicenter prospective studies incorporating comprehensive clinical and laboratory assessments are warranted to validate our findings and further clarify the role of diabetes‐specific factors in fall risk among older adults.

## 5. Conclusion

In conclusion, our findings demonstrate a considerable fall burden among community‐dwelling older adults with T2DM in urban Shanghai, with associated factors spanning physical, psychological, and behavioral domains. These findings underscore the need to expand diabetes care beyond traditional glucose‐centric approaches and advocate for the integration of multidomain fall screening in this population. Particular attention should be given to elderly individuals with poor balance, depression or activity restriction due to fear of falling. Screening for these factors may facilitate early identification of those at risk and inform targeted assessment approaches.

## Author Contributions

Methodology and investigation: Zhenping Lu, Hui Gao, Huiyao Zhu, Dong Chen, Yinzhu Zhuang, Lei Zhang, Li Yu, Qinghua Xia, and Yu Jiang. Data curation, formal analysis, and writing—original draft: Zhenping Lu. Conceptualization, supervision, and writing—review and editing: Yu Jiang and Qinghua Xia.

## Funding

This study was supported by the Shanghai New Three‐year Action Plan for Public Health (GWVI‐11.2‐XD05) and The Master and Doctoral‐level innovative talent program on “Multi‐Dimensional Diabetes Management” (RCJD2021S06).

## Disclosure

All authors have read and approved the final version of the manuscript. Yu Jiang had full access to all of the data in this study and takes complete responsibility for the integrity of the data and the accuracy of the data analysis.

## Ethics Statement

This study was approved by the Ethics Committee of the Changning District Center for Disease Control and Prevention, Shanghai (No.: 2023‐08). All participants received comprehensive information about the study and provided written informed consent prior to enrollment. We confirm that all methods were performed in strict accordance with the applicable guidelines and regulations.

## Conflicts of Interest

The authors declare no conflicts of interest.

## Supporting Information

Additional supporting information can be found online in the Supporting Information section.

## Supporting information


**Supporting Information 1** Table S1: Self‐designed questionnaire for baseline data collection. It covers participant demographics, medical conditions, daily behaviors, home environmental factors associated with falls, and physical measurements. Table S2: Baseline characteristics and potential associated factors for falls among elderly with T2DM, presented overall and by sex. This table includes variables that showed no significant sex differences or were of relatively lower priority to the main study population. Table S3: Comparison of potential associated factors across different fall categories. This table presents variables that were not statistically significant in relation to falls.

**Supporting Information 2**  

## Data Availability

The data that support the findings of this study are available from the corresponding author upon reasonable request.
