# Supplementary material for: Factors Associated With Falls Among the Elderly With Type 2 Diabetes in Urban Shanghai: A Cross‐Sectional Study
Source: J Diabetes Res. 2026 Sep 24;2026:8624656. doi: 10.1155/jdr/8624656 (PMC13606027; doi:10.1155/jdr/8624656)
Supplement: Supplementary file 1 [file JDR-2026-8624656-s001.docx]

Factors associated with falls among the elderly with type 2 diabetes in urban Shanghai: a cross-sectional study (Supplementary materials)

Table S1 Self-designed questionnaire for baseline data collection

| **Section 1:** **Demographics** | | | | |
| --- | --- | --- | --- | --- |
| Questions | | | Options | |
| Gender | | | A. Male | |
|  |  |  | B. Female | |
| Age | | | years old | |
| Marital Status | | | A. Married | |
|  |  |  | B. Unmarried | |
|  |  |  | C. Divorced or Widowed | |
|  |  |  | D. Marital Status Not Specified | |
| Educational Level | | | A. Illiterate/Semi-illiterate | |
|  |  |  | B. Primary School | |
|  |  |  | C. Junior High School | |
|  |  |  | D. High School or Secondary Technical School | |
|  |  |  | E. Junior College | |
|  |  |  | F. University or Above | |
| Cohabitation Status | | | A. Living alone | |
|  |  |  | B. Living only with spouse | |
|  |  |  | C. Living with children | |
|  |  |  | D. Other | |
| Care Arrangement | | | A. Self-care | |
|  |  |  | B. Cared for by relatives | |
|  |  |  | C. Cared for by professional caregiver/nanny | |
|  |  |  | D. Other | |
| Total Annual Household Income in the Past Year (yuan) | | | A. < 10,000 | |
|  |  |  | B. 10,000-50,000 | |
|  |  |  | C. 60,000-100,000 | |
|  |  |  | D. 110,000-200,000 | |
|  |  |  | E. > 200,000 | |
| **Section 2:** **Medical Conditions** | | | | |
| Questions | | Options | | |
| Do you have any of the following cardiovascular or cerebrovascular diseases | | A. Hypertension | | |
|  |  | B. Heart Disease | | |
|  |  | C. Stroke (Cerebral Infarction, Cerebral Hemorrhage) | | |
|  |  | D. Other | | |
| Do you have any of the following endocrine, nutritional, or metabolic diseases | | A. Diabetes, Year of Diagnosis: _______ | | |
|  |  | B. Osteoporosis | | |
|  |  | C. Arthritis (Rheumatoid/Osteoarthritis) | | |
|  |  | D. Other | | |
| Do you have any of the following neurological diseases | | A. Cerebellar Disorders | | |
|  |  | B. Peripheral Neuropathy | | |
|  |  | C. Parkinson's Disease/Parkinsonian Syndromes | | |
|  |  | D. Epilepsy | | |
|  |  | E. Other | | |
| Do you have any of the following respiratory diseases | | A. Chronic Obstructive Pulmonary Disease (COPD) | | |
|  |  | B. Asthma | | |
|  |  | C. Tuberculosis | | |
|  |  | D. Other | | |
| Do you have any of the following musculoskeletal diseases | | A. Bone Spurs (Osteophytes) | | |
|  |  | B. Cervical Spondylosis | | |
|  |  | C. Lumbar Disc Herniation | | |
|  |  | D. Other | | |
| Do you have any of the following mental disorders | | A. Depression | | |
|  |  | B. Dementia | | |
|  |  | C. Other | | |
| Do you have any tumors | | A. No | | |
|  |  | B. Yes, please specify: ________________ | | |
| Do you have any of the following eye diseases | | A. Cataracts | | |
|  |  | B. Glaucoma | | |
|  |  | C. High Myopia (Severe Nearsightedness) | | |
|  |  | D. Other | | |
| Do you have any of the following ear diseases | | A. Otitis Media | | |
|  |  | B. Vestibular Disorders | | |
|  |  | C. Hearing Loss/Deafness | | |
|  |  | D. Other | | |
| In the past year, how has your blood glucose control been | | A. Well-controlled, largely achieving ideal levels | | |
|  |  | B. Well-controlled most of the time | | |
|  |  | C. Poorly controlled | | |
| Have you been diagnosed with diabetic nephropathy | | A. Yes | | |
|  |  | B. No | | |
| Have you been diagnosed with diabetic eye disease | | A. Yes | | |
|  |  | B. No | | |
| Have you been diagnosed with diabetic neuropathy | | A. Yes | | |
|  |  | B. No | | |
| Have you been diagnosed with diabetic foot | | A. Yes | | |
|  |  | B. No | | |
| Have you ever experienced any of the following acute diabetic complications (diabetic ketoacidosis, hyperosmolar hyperglycemic state, diabetic lactic acidosis, hypoglycemia) | | A. Yes | | |
|  |  | B. No | | |
| In the past year, have you frequently felt dizzy | | A. Yes | | |
|  |  | B. No | | |
| In the past week, on how many days did you feel physically fatigued or have difficulty walking | | ______ days | | |
| Do you experience tingling or numbness in your limbs | | A. Yes | | |
|  |  | B. No | | |
| Do you feel unsteady while standing or walking | | A. Yes | | |
|  |  | B. No | | |
| Do you limit or reduce your activities due to fear of falling | | A. Yes | | |
|  |  | B. No | | |
| Are you currently using any glucose-lowering medications | | A. Yes, please specify: ________________ | | |
|  |  | B. No | | |
| Are you currently taking any sedative or hypnotic medications | | A. Yes, please specify: ________________ | | |
|  |  | B. No | | |
| Are you currently taking 4 or more medications simultaneously | | A. Yes | | |
|  |  | B. No | | |
| **Section 3:** **Daily Behaviors** | | | | |
| Questions | | Options | | |
| In the past year, have you experienced a fall | | A. Yes, _______ times | | |
|  |  | B. No | | |
| Use of cane/walking aid | | A. Yes | | |
|  |  | B. No | | |
| Do you currently smoke | | A. Yes, _________ cigarettes per day | | |
|  |  | B. No | | |
| Your average daily sleep duration | | Night: ______ hours, Nap: ______ hours | | |
| Your usual number of nighttime awakenings per night | | ______ times | | |
| Engages in habitual physical exercise (total weekly exercise time exceeds 150 minutes) | | A. Yes | | |
|  |  | B. No | | |
| Dietary Habits | | A. Balanced mix of meat and vegetables | | |
|  |  | B. Mainly Vegetarian | | |
|  |  | C. Mainly Meat-Based | | |
| **Section 4:** **Home Environmental Factors Associated with Falls** | | | | |
| Category | Identified Problems / Hazards | | | Location  (①Living Room②Bedroom③Kitchen④Bathroom⑤Other) |
| Lighting | No lighting installed in areas where the elderly are active | | |  |
|  | Insufficient, excessive, glaring, or flickering lighting | | |  |
|  | Inconveniently located light switches when entering a room or getting up at night | | |  |
|  | Not using night lights, flashlights, or other nighttime lighting aids | | |  |
| Slippery Floors | Slippery floors due to water, oil, or other spills | | |  |
|  | Flooring or surface materials that are excessively smooth | | |  |
|  | Slippery floors caused by waxing | | |  |
| Rugs and Mats | Curled or turned-up edges of rugs or mats | | |  |
|  | Loose or easily sliding rugs or mats | | |  |
| Obstructions | Furniture, clutter, or temporarily placed items obstructing hallways and walkways | | |  |
|  | Electrical cords or ropes extending across hallways and walkways | | |  |
| Furniture Selection and Arrangement | Sofas, chairs, or beds are too high or too low | | |  |
|  | The toilet seat is too high or too low | | |  |
|  | No arm-rests or support aids available when getting up from sofas, chairs, or beds | | |  |
|  | Furniture incorporates large amounts of glass, fragile materials, or sharp edges as decorative elements | | |  |
|  | Unstable furniture, such as wheeled chairs or tables, or poorly assembled tables | | |  |
|  | Furniture arrangement hinders movement within the room, such as blocking pathways or forcing unnecessary detours | | |  |
|  | Cabinets, drawers, or shelves for storing food, condiments, or daily necessities are positioned too high or too low | | |  |
|  | Clothes-drying racks are unsuitable for older adults, requiring them to lean out over a balcony or stand on a stool to hang laundry | | |  |
|  | If shoe removal is required upon entry, there is no seating available at the entryway for older adults to use | | |  |
| Support Aids | No grab bars beside the toilet, bathtub, or in the shower area | | |  |
|  | No handrails where indoor steps or level changes exist | | |  |
| Hallways and Stairs | Burnt-out light bulbs or insufficient lighting in hallways | | |  |
|  | Clutter piled along hallway walls | | |  |
|  | Stair edges are not clearly visible | | |  |
|  | Damaged or broken stair steps | | |  |
|  | No handrails along the staircase | | |  |
|  | In buildings without elevators, no resting seat is provided for older adults on stair landings | | |  |
| Pets | Pets do not have a designated resting area within the home | | |  |
|  | Pets are not easily visible within the home environment | | |  |
| Other | Please specify: | | |  |
| **Section 5:** **Physical Measurement** | | | | |
| Indexes | | Results | | |
| Height | | ____________ cm | | |
| Weight | | ____________ kg | | |
| Waist Circumference | | ____________ cm | | |
| Calf Circumference | | ____________ cm | | |

Table S2 Baseline characteristics and potential associated factors for falls among the elderly with T2DM, overall and by sex

| **Variables** | **Overall**  **(N = 842)** | **Male**  **(N = 333)** | **Female**  **(N = 509)** | ***χ*^2^** | ***P* value** |
| --- | --- | --- | --- | --- | --- |
| **Marital Status** |  |  |  | 1.525 | 0.677 |
| Married | 613(72.8%) | 244(73.3%) | 369(72.5%) |  |  |
| Unmarried | 20(2.4%) | 10(3.0%) | 10(2.0%) |  |  |
| Divorced or Widowed | 33(3.9%) | 11(3.3%) | 22(4.3%) |  |  |
| Marital Status Not Specified | 176(20.9%) | 68(20.4%) | 108(21.2%) |  |  |
| **Total Annual Household Income in the Past Year (yuan)** |  |  |  | 6.342 | 0.096 |
| Very low (< 10,000) | 48(5.7%) | 12(3.6%) | 36(7.1%) |  |  |
| Low (10,000-100,000) | 414(49.2%) | 174(52.3%) | 240(47.2%) |  |  |
| Moderate (100,000-200,000) | 331(39.3%) | 125(37.5%) | 206(40.5%) |  |  |
| High (> 200,000) | 49(5.8%) | 22(6.6%) | 27(5.3%) |  |  |
| **Environmental Hazard(s) at Home** |  |  |  | 0.080 | 0.778 |
| No | 227(27%) | 88(26.4%) | 139(27.3%) |  |  |
| Yes | 615(73%) | 245(73.6%) | 370(72.7%) |  |  |
| **Glycemic Control Status** |  |  |  | 2.563 | 0.109 |
| Good | 765(90.9%) | 296(88.9%) | 469(92.1%) |  |  |
| Poor | 77(9.1%) | 37(11.1%) | 40(7.9%) |  |  |
| **Acute Diabetic Complications** |  |  |  | 0.004 | 0.949 |
| Yes | 45(5.3%) | 18(5.4%) | 27(5.3%) |  |  |
| No | 797 (94.7%) | 315(94.6%) | 482(94.7%) |  |  |
| **Diabetic Nephropathy** |  |  |  | 0.662 | 0.416 |
| Yes | 37 (4.4%) | 17(5.1%) | 20(3.9%) |  |  |
| No | 805(95.6%) | 316(94.9%) | 489(96.1%) |  |  |
| **Diabetic Eye Disease** |  |  |  | 0.315 | 0.574 |
| Yes | 37 (4.4%) | 13(3.9%) | 24(4.7%) |  |  |
| No | 805(95.6%) | 320(96.1%) | 485(95.3%) |  |  |
| **Diabetic Neuropathy** |  |  |  | 1.483 | 0.223 |
| Yes | 21(2.5%) | 11(3.3%) | 10(2.0%) |  |  |
| No | 821(97.5%) | 322(96.7%) | 499(98.0%) |  |  |
| **Diabetic Foot Disease** |  |  |  | 0.408 | 0.523 |
| Yes | 24(2.9%) | 11(3.3%) | 13(2.6%) |  |  |
| No | 818(97.1%) | 322(96.7%) | 496(97.4%) |  |  |
| **Insulin Use** |  |  |  | 1.484 | 0.223 |
| No | 728(86.5%) | 282(84.7%) | 446(87.6%) |  |  |
| Yes | 114(13.5%) | 51(15.3%) | 63(12.4%) |  |  |
| **Thiazolidinedione (TZD) Use** |  |  |  | 0.046 | 0.830 |
| No | 818(97.1%) | 323(97.0%) | 495(97.2%) |  |  |
| Yes | 24(2.9%) | 10(3.0%) | 14(2.8%) |  |  |
| **Sulfonylurea Use** |  |  |  | 0.010 | 0.919 |
| No | 760(90.3%) | 301(90.4%) | 459(90.2%) |  |  |
| Yes | 82(9.7%) | 32(9.6%) | 50(9.8%) |  |  |
| **Use of Sedative-Hypnotic or Psychotropic Medications** |  |  |  | 0.010 | 0.922 |
| No | 742(88.1%) | 293(88.0%) | 449(88.2%) |  |  |
| Yes | 100(11.9%) | 40(12.0%) | 60(11.8%) |  |  |
| **Frequent Dizziness** |  |  |  | 2.907 | 0.088 |
| No | 623(74.0%) | 257(77.2%) | 366(71.9%) |  |  |
| Yes | 219(26.0%) | 76(22.8%) | 143(28.1%) |  |  |
| **Persistent Fatigue** |  |  |  | 0.000 | 0.990 |
| No | 741(88.0%) | 293(88.0%) | 448(88.0%) |  |  |
| Yes | 101(12.0%) | 40(12.0%) | 61(12.0%) |  |  |
| **History of Limb Paresthesia** |  |  |  | 2.905 | 0.088 |
| No | 537(63.8%) | 224(67.3%) | 313(61.5%) |  |  |
| Yes | 305(36.2%) | 109(32.7%) | 196(38.5%) |  |  |
| **Body Weight Status** |  |  |  | 7.248 | 0.064 |
| Underweight | 37(4.4%) | 14(4.2%) | 23(4.5%) |  |  |
| Normal weight | 390(46.3%) | 136(40.8%) | 254(49.9%) |  |  |
| Overweight | 315(37.4%) | 138(41.4%) | 177(34.8%) |  |  |
| Obesity | 100(11.9%) | 45(13.5%) | 55(10.8%) |  |  |
| **Increased Risk of Sarcopenia** |  |  |  | 3.678 | 0.055 |
| No | 541(64.3%) | 227(68.2%) | 314(61.7%) |  |  |
| Yes | 301(35.7%) | 106(31.8%) | 195(38.3%) |  |  |
| **Timed Up and Go Test (Time > 15s)** |  |  |  | 0.134 | 0.715 |
| No | 792(94.1%) | 312(93.7%) | 480(94.3%) |  |  |
| Yes | 50(5.9%) | 21(6.3%) | 29(5.7%) |  |  |
| **Five Times Sit-to-Stand (Time > 15s)** |  |  |  | 1.161 | 0.281 |
| No | 717(85.2%) | 289(86.8%) | 428(84.1%) |  |  |
| Yes | 125(14.8%) | 44(13.2%) | 81(15.9%) |  |  |
| **Balance Test Score** |  |  |  | 0.091 | 0.763 |
| ≥ 17 | 658(78.1%) | 262(78.7%) | 396(77.8%) |  |  |
| < 17 | 184(21.9%) | 71(21.3%) | 113(22.2%) |  |  |
| **Balanced Diet** |  |  |  | 0.025 | 0.875 |
| No | 131(15.6%) | 51(15.3%) | 80(15.7%) |  |  |
| Yes | 711(84.4%) | 282(84.7%) | 429(84.3%) |  |  |
| **Frequent Nocturia ( ≥ 2 times/night)** |  |  |  | 2.377 | 0.123 |
| No | 490(58.2%) | 183(55.0%) | 307(60.3%) |  |  |
| Yes | 352(41.8%) | 150(45.0%) | 202(39.7%) |  |  |
| **Daily Use of Walking Aids** |  |  |  | 0.726 | 0.394 |
| No | 784(93.1%) | 307(92.2%) | 477(93.7%) |  |  |
| Yes | 58(6.9%) | 26(7.8%) | 32(6.3%) |  |  |

Note: Acute diabetic complications include diabetic ketoacidosis, hyperosmolar hyperglycemic state, diabetic lactic acidosis, and hypoglycemia.

Table S3 Comparison of potential associated factors across different fall categories

| **Variables** | **Total Fallers**  **(N = 239)** | **Non-fallers**  **(N = 603)** | ***χ*^2^** | ***P* value** | **Non- recurrent Fallers (N = 801)** | **Recurrent Fallers**  **(N = 41)** | ***χ*^2^** | ***P* value** |
| --- | --- | --- | --- | --- | --- | --- | --- | --- |
| **Demographic and Socioeconomic Characteristics** | | | | | | | | |
| **Gender** |  |  | 1.775 | 0.183 |  |  | 0.066 | 0.797 |
| Male | 86(36.0%) | 247(41.0%) |  |  | 316(39.5%) | 17(41.5%) |  |  |
| Female | 153(64.0%) | 356(59.0%) |  |  | 485(60.5%) | 24(58.5%) |  |  |
| **Age (years)** |  |  | 1.052 | 0.591 |  |  | -^a^ | 0.391 |
| 60-69 | 74(31.0%) | 209(34.7%) |  |  | 268(33.5%) | 15(36.6%) |  |  |
| 70-79 | 136(56.9%) | 324(53.7%) |  |  | 441(55.1%) | 19(46.3%) |  |  |
| ≥ 80 | 29(12.1%) | 70(11.6%) |  |  | 92(11.5%) | 7(17.1%) |  |  |
| **Marital Status** |  |  | 5.076 | 0.166 |  |  | -^a^ | 0.771 |
| Married | 187(78.2%) | 426(70.6%) |  |  | 584(72.9%) | 29(70.7%) |  |  |
| Unmarried | 5(2.1%) | 15(2.5%) |  |  | 20(2.5%) | 0(0%) |  |  |
| Divorced or Widowed | 7(2.9%) | 26(4.3%) |  |  | 31(3.9%) | 2(4.9%) |  |  |
| Marital Status Not Specified | 40(16.7%) | 136(22.6%) |  |  | 166(20.7%) | 10(24.4%) |  |  |
| **Educational Level** |  |  | 3.146 | 0.370 |  |  | -^a^ | 0.757 |
| Primary school or below | 26(10.9%) | 46(7.6%) |  |  | 67(8.4%) | 5(12.2%) |  |  |
| Junior high school | 100(41.8%) | 241(40%) |  |  | 324(40.4%) | 17(41.5%) |  |  |
| Senior high school or technical secondary school | 81(33.9%) | 226(37.5%) |  |  | 294(36.7%) | 13(31.7%) |  |  |
| Junior college or above | 32(13.4%) | 90(14.9%) |  |  | 116(14.5%) | 6(14.6%) |  |  |
| **Living Alone** |  |  | 0.716 | 0.398 |  |  | -^a^ | 0.215 |
| No | 215(90%) | 530(87.9%) |  |  | 706(88.1%) | 39(95.1%) |  |  |
| Yes | 24(10%) | 73(12.1%) |  |  | 95(11.9%) | 2(4.9%) |  |  |
| **Total Annual Household Income in the Past Year (yuan)** | | | 7.768 | 0.051 |  |  | -^a^ | 0.374 |
| Very low (< 10,000) | 11(4.6%) | 37(6.1%) |  |  | 48(6.0%) | 0(0%) |  |  |
| Low (10,000-100,000) | 122(51.0%) | 292(48.4%) |  |  | 392(48.9%) | 22(53.7%) |  |  |
| Moderate (100,000-200,000) | 100(41.8%) | 231(38.3%) |  |  | 313(39.1%) | 18(43.9%) |  |  |
| High (> 200,000) | 6(2.5%) | 43(7.1%) |  |  | 48(6.0%) | 1(2.4%) |  |  |
| **Environmental Hazard(s) at Home** |  |  | 0.876 | 0.349 |  |  | 0.493 | 0.482 |
| No | 59(24.7%) | 168(27.9%) |  |  | 214(26.7%) | 13(31.7%) |  |  |
| Yes | 180(75.3%) | 435(72.1%) |  |  | 587(73.3%) | 28(68.3%) |  |  |
| **Clinical and Comorbidity-related Factors** | | | | | | | | |
| **Duration of Diabetes (years)** |  |  | 1.532 | 0.465 |  |  | 1.961 | 0.375 |
| < 5 | 79(33.1%) | 211(35.0%) |  |  | 278(34.7%) | 12(29.3%) |  |  |
| 5-10 | 66(27.6%) | 182(30.2%) |  |  | 238(29.7%) | 10(24.4%) |  |  |
| ≥ 10 | 94 (39.3%) | 210 (34.8%) |  |  | 285 (35.6%) | 19 (46.3%) |  |  |
| **Glycemic Control Status** |  |  | 0.323 | 0.570 |  |  | -^a^ | 1.000 |
| Good | 215(90%) | 550(91.2%) |  |  | 727(90.8%) | 38(92.7%) |  |  |
| Poor | 24(10%) | 53(8.8%) |  |  | 74(9.2%) | 3(7.3%) |  |  |
| **Acute Diabetic Complications** |  |  | 1.203 | 0.273 |  |  | -^a^ | 0.061 |
| Yes | 16(6.7%) | 29(4.8%) |  |  | 40(5.0%) | 5(12.2%) |  |  |
| No | 223(93.3%) | 574(95.2%) |  |  | 761(95.0%) | 36(87.8%) |  |  |
| **Diabetic Nephropathy** |  |  | 0.312 | 0.577 |  |  | -^a^ | 1.000 |
| Yes | 12(5.0%) | 25(4.1%) |  |  | 36(4.5%) | 1(2.4%) |  |  |
| No | 227(95.0%) | 578(95.9%) |  |  | 765(95.5%) | 40(97.6%) |  |  |
| **Diabetic Eye Disease** |  |  | 1.701 | 0.192 |  |  | -^a^ | 0.418 |
| Yes | 14(5.9%) | 23(3.8%) |  |  | 34(4.2%) | 3(7.3%) |  |  |
| No | 225(94.1%) | 580(96.2%) |  |  | 767(95.8%) | 38(92.7%) |  |  |
| **Diabetic Neuropathy** |  |  | 0.259 | 0.610 |  |  | -^a^ | 0.077 |
| Yes | 7(2.9%) | 14(2.3%) |  |  | 18(2.2%) | 3(7.3%) |  |  |
| No | 232(97.1%) | 589(97.7%) |  |  | 783(97.8%) | 38(92.7%) |  |  |
| **Diabetic Foot Disease** |  |  | 0.139 | 0.709 |  |  | -^a^ | 1.000 |
| Yes | 6(2.5%) | 18(3.0%) |  |  | 23(2.9%) | 1(2.4%) |  |  |
| No | 233(97.5%) | 585(97.0%) |  |  | 778(97.1%) | 40(97.6%) |  |  |
| **Presence of Multimorbidity (≥ 5 conditions)** | | | 1.595 | 0.207 |  |  | 0.937 | 0.333 |
| No | 188(78.7%) | 497(82.4%) |  |  | 654(81.6%) | 31(75.6%) |  |  |
| Yes | 51(21.3%) | 106(17.6%) |  |  | 147(18.4%) | 10(24.4%) |  |  |
| **Medication-related Factors** | | | | | | | | |
| **Insulin Use** |  |  | 1.075 | 0.300 |  |  | 0.067 | 0.796 |
| No | 202(84.5%) | 526(87.2%) |  |  | 692(86.4%) | 36(87.8%) |  |  |
| Yes | 37(15.5%) | 77(12.8%) |  |  | 109(13.6%) | 5(12.2%) |  |  |
| **Thiazolidinedione (TZD) Use** |  |  | 0.693 | 0.405 |  |  | -^a^ | 0.328 |
| No | 234(97.9%) | 584(96.8%) |  |  | 779(97.3%) | 39(95.1%) |  |  |
| Yes | 5(2.1%) | 19(3.2%) |  |  | 22(2.7%) | 2(4.9%) |  |  |
| **Sulfonylurea Use** |  |  | 0.198 | 0.657 |  |  | -^a^ | 0.789 |
| No | 214(89.5%) | 546(90.5%) |  |  | 722(90.1%) | 38(92.7%) |  |  |
| Yes | 25(10.5%) | 57(9.5%) |  |  | 79(9.9%) | 3(7.3%) |  |  |
| **Use of** **Sedative-Hypnotic or Psychotropic Medications** | | | 1.189 | 0.276 |  |  | -^a^ | 0.318 |
| No | 206(86.2%) | 536(88.9%) |  |  | 708(88.4%) | 34(82.9%) |  |  |
| Yes | 33(13.8%) | 67(11.1%) |  |  | 93 (11.6%) | 7(17.1%) |  |  |
| **Polypharmacy** **(≥ 4 Medications)** |  |  | 1.569 | 0.210 |  |  | 1.090 | 0.296 |
| No | 167(69.9%) | 447(74.1%) |  |  | 587(73.3%) | 27(65.9%) |  |  |
| Yes | 72(30.1%) | 156(25.9%) |  |  | 214(26.7%) | 14(34.1%) |  |  |
| **Physical Function and Mobility Factors** | | | | | | | | |
| **Body Weight Status** |  |  | 0.982 | 0.806 |  |  | -^a^ | 0.350 |
| Underweight | 9(3.8%) | 28(4.6%) |  |  | 37(4.6%) | 0(0%) |  |  |
| Normal weight | 109(45.6%) | 281(46.6%) |  |  | 366(45.7%) | 24(58.5%) |  |  |
| Overweight | 89(37.2%) | 226(37.5%) |  |  | 302(37.7%) | 13(31.7%) |  |  |
| Obesity | 32(13.4%) | 68(11.3%) |  |  | 96(12.0%) | 4(9.8%) |  |  |
| **Central Obesity** |  |  | 1.585 | 0.208 |  |  | 0.267 | 0.605 |
| No | 82(34.3%) | 235(39%) |  |  | 300(37.5%) | 17(41.5%) |  |  |
| Yes | 157(65.7%) | 368(61%) |  |  | 501(62.5%) | 24(58.5%) |  |  |
| **Increased Risk of Sarcopenia** |  |  | 0.752 | 0.386 |  |  | 3.187 | 0.074 |
| No | 159(66.5%) | 382(63.3%) |  |  | 520(64.9%) | 21(51.2%) |  |  |
| Yes | 80(33.5%) | 221(36.7%) |  |  | 281(35.1%) | 20(48.8%) |  |  |
| **Timed Up and Go Test (Time > 15s)** | | | 3.528 | 0.060 |  |  | -^a^ | 1.000 |
| No | 219(91.6%) | 573(95%) |  |  | 753(94.0%) | 39(95.1%) |  |  |
| Yes | 20(8.4%) | 30(5%) |  |  | 48(6.0%) | 2(4.9%) |  |  |
| **Five Times Sit-to-Stand (Time > 15s)** | |  | 1.964 | 0.161 |  |  | 0.169 | 0.681 |
| No | 197(82.4%) | 520(86.2%) |  |  | 683(85.3%) | 34(82.9%) |  |  |
| Yes | 42(17.6%) | 83(13.8%) |  |  | 118(14.7%) | 7(17.1%) |  |  |
| **Psychological and Cognitive Factors** | | | | | | | | |
| **Presence of Cognitive Impairment** |  |  | 0.002 | 0.966 |  |  | 0.015 | 0.903 |
| Normal cognition | 155(64.9%) | 392(65.0%) |  |  | 520(64.9%) | 27(65.9%) |  |  |
| Cognitive impairment | 84(35.1%) | 211(35.0%) |  |  | 281(35.1%) | 14(34.1%) |  |  |
| **Lifestyle and Behavioral Factors** | | | | | | | | |
| **Smoking** |  |  | 0.988 | 0.320 |  |  | -^a^ | 0.792 |
| No | 218(91.2%) | 536(88.9%) |  |  | 716(89.4%) | 38(92.7%) |  |  |
| Yes | 21(8.8%) | 67(11.1%) |  |  | 85(10.6%) | 3(7.3%) |  |  |
| **Balanced Diet** |  |  | 2.066 | 0.151 |  |  | 0.513 | 0.474 |
| No | 44(18.4%) | 87(14.4%) |  |  | 123(15.4%) | 8(19.5%) |  |  |
| Yes | 195(81.6%) | 516(85.6%) |  |  | 678(84.6%) | 33(80.5%) |  |  |

Note: ᵃ Fisher’s exact test was used for variables with expected cell counts <5.
