# Supplementary material for: Factors Associated With Falls Among the Elderly With Type 2 Diabetes in Urban Shanghai: A Cross‐Sectional Study
Source: J Diabetes Res. 2026 Sep 24;2026:8624656. doi: 10.1155/jdr/8624656 (PMC13606027; doi:10.1155/jdr/8624656)
Supplement: Supplementary file 2 [file JDR-2026-8624656-s002.doc]

STROBE Statement—Checklist of items that should be included in reports of ***cross-sectional studies***

| **Section** | Item No | Recommendation | Where reported |
| --- | --- | --- | --- |
| **Title and abstract** | 1 | (*a*) Indicate the study’s design with a commonly used term in the title or the abstract | Page 1, line 1-2 in revised manuscript-clean version |
|  |  | (*b*) Provide in the abstract an informative and balanced summary of what was done and what was found | Page 1, line 22-37 |
| Introduction | | |  |
| Background/rationale | 2 | Explain the scientific background and rationale for the investigation being reported | Page 2, line 41-54 |
| Objectives | 3 | State specific objectives, including any prespecified hypotheses | Page 2, line 54-56 |
| Methods | | |  |
| Study design | 4 | Present key elements of study design early in the paper | Page 2, line 60 |
| Setting | 5 | Describe the setting, locations, and relevant dates, including periods of recruitment, exposure, follow-up, and data collection | Page 2, line 60-61 |
| Participants | 6 | (*a*) Give the eligibility criteria, and the sources and methods of selection of participants | Page 2, line 61-71 |
| Variables | 7 | Clearly define all outcomes, exposures, predictors, potential confounders, and effect modifiers. Give diagnostic criteria, if applicable | Page 3, line 88-100 |
| Data sources/ measurement | 8* | For each variable of interest, give sources of data and details of methods of assessment (measurement). Describe comparability of assessment methods if there is more than one group | Page 3, line 101-116 |
| Bias | 9 | Describe any efforts to address potential sources of bias | Page 2, line 69-71 |
| Study size | 10 | Explain how the study size was arrived at | Page 3, 71-75 |
| Quantitative variables | 11 | Explain how quantitative variables were handled in the analyses. If applicable, describe which groupings were chosen and why | Page 3, line 88-100 |
| Statistical methods | 12 | (*a*) Describe all statistical methods, including those used to control for confounding | Page 3-4,  line 118-129 |
|  |  | (*b*) Describe any methods used to examine subgroups and interactions | -^a^ |
|  |  | (*c*) Explain how missing data were addressed | Page 4, line 130-134 |
|  |  | (*d*) If applicable, describe analytical methods taking account of sampling strategy | -^b^ |
|  |  | (*e*) Describe any sensitivity analyses | -^c^ |
| Results | | |  |
| Participants | 13* | (a) Report numbers of individuals at each stage of study—eg numbers potentially eligible, examined for eligibility, confirmed eligible, included in the study, completing follow-up, and analysed | Page 2, line 71-74 |
|  |  | (b) Give reasons for non-participation at each stage | Page 2, line 71-73 |
|  |  | (c) Consider use of a flow diagram | Page 4, Figure 1 |
| Descriptive data | 14* | (a) Give characteristics of study participants (eg demographic, clinical, social) and information on exposures and potential confounders | Page 4-6,  line 142-158 |
|  |  | (b) Indicate number of participants with missing data for each variable of interest | Page 4, line 130-134^d^ |
| Outcome data | 15* | Report numbers of outcome events or summary measures | Page 6, line 161-162 |
| Main results | 16 | (*a*) Give unadjusted estimates and, if applicable, confounder-adjusted estimates and their precision (eg, 95% confidence interval). Make clear which confounders were adjusted for and why they were included | Page 7-9,  line 178-202 |
|  |  | (*b*) Report category boundaries when continuous variables were categorized | Page 5-6, table 1  Page 6-7, table 2 |
|  |  | (*c*) If relevant, consider translating estimates of relative risk into absolute risk for a meaningful time period | Not applicable |
| Other analyses | 17 | Report other analyses done—eg analyses of subgroups and interactions, and sensitivity analyses | - |
| Discussion | | |  |
| Key results | 18 | Summarise key results with reference to study objectives | Page 9, line 206-210 |
| Limitations | 19 | Discuss limitations of the study, taking into account sources of potential bias or imprecision. Discuss both direction and magnitude of any potential bias | Page 10,  line 265-272 |
| Interpretation | 20 | Give a cautious overall interpretation of results considering objectives, limitations, multiplicity of analyses, results from similar studies, and other relevant evidence | Page 9-10,  line 211-261 |
| Generalisability | 21 | Discuss the generalisability (external validity) of the study results | Page 10, line 269-270; line 278-280 |
| Other information | | |  |
| Funding | 22 | Give the source of funding and the role of the funders for the present study and, if applicable, for the original study on which the present article is based | Page 11,  line 311-313 |

*Give information separately for exposed and unexposed groups.

**Note:** An Explanation and Elaboration article discusses each checklist item and gives methodological background and published examples of transparent reporting. The STROBE checklist is best used in conjunction with this article (freely available on the Web sites of PLoS Medicine at http://www.plosmedicine.org/, Annals of Internal Medicine at http://www.annals.org/, and Epidemiology at http://www.epidem.com/). Information on the STROBE Initiative is available at [www.strobe-statement.org](http://www.strobe-statement.org/).

a: No subgroup or interaction analyses were conducted, as these were not prespecified in the study protocol and the primary aim was to estimate the overall association.

b: Given the simple random sampling design, no sampling weights or clustering adjustments were applied.

c: Sensitivity analyses were not performed because the results were sufficiently robust, with minimal missing data, validated variable definitions, and a prespecified analytical plan.

d: Missing data for each variable were detailed in the Methods section; all analyses were performed on complete-case data with no missing values.
